# A Point-Line-Area Paradigm: 3D Printing for Next-Generation Health Monitoring Sensors

**DOI:** 10.3390/s25185777

**Published:** 2025-09-16

**Authors:** Mei Ming, Xiaohong Yin, Yinchen Luo, Bin Zhang, Qian Xue

**Affiliations:** 1 School of Mechanical Engineering, Zhejiang University, Hangzhou 310058, China; 12425041@zju.edu.cn (M.M.); 11825040@zju.edu.cn (X.Y.); 2 College of Electrical Engineering, Zhejiang University, Hangzhou 310058, China; luoyichen@zju.edu.cn; 3 Institute of Advanced Machines, Zhejiang University, Hangzhou 310058, China

**Keywords:** 3D printing, health monitoring, sensors, multifunctional sensing, additive manufacturing, personalized healthcare

## Abstract

Three-dimensional printing technology is fundamentally reshaping the design and fabrication of health monitoring sensors. While it holds great promise for achieving miniaturization, multi-material integration, and personalized customization, the lack of a clear selection framework hinders the optimal matching of printing technologies to specific sensor requirements. This review presents a classification framework based on existing standards and specifically designed to address sensor-related requirements, categorizing 3D printing technologies into point-based, line-based, and area-based modalities according to their fundamental fabrication unit. This framework directly bridges the capabilities of each modality, such as nanoscale resolution, multi-material versatility, and high-throughput production, with the critical demands of modern health monitoring sensors. We systematically demonstrate how this approach guides technology selection: Point-based methods (e.g., stereolithography, inkjet) enable micron-scale features for ultra-sensitive detection; line-based techniques (e.g., Direct Ink Writing, Fused Filament Fabrication) excel in multi-material integration for creating complex functional devices such as sweat-sensing patches; and area-based approaches (e.g., Digital Light Processing) facilitate rapid production of sensor arrays and intricate structures for applications like continuous glucose monitoring. The point–line–area paradigm offers a powerful heuristic for designing and manufacturing next-generation health monitoring sensors. We also discuss strategies to overcome existing challenges, including material biocompatibility and cross-scale manufacturing, through the integration of AI-driven design and stimuli-responsive materials. This framework not only clarifies the current research landscape but also accelerates the development of intelligent, personalized, and sustainable health monitoring systems.

## 1. Introduction

The wide application of sensors has permeated every aspect of human life, from industrial production and environmental protection to the growing demand for human health monitoring in recent years. With global aging and the earlier onset of diseases, health management is undergoing a transformation from “treatment-centered” to “prevention-focused”. According to the World Health Organization (WHO), the proportion of the global population aged 60 and above is projected to increase from 12 percent to 22 percent by 2050, reaching 1.4 billion by then [1]. By 2020, the number of people aged 60 and above in China had climbed to 265 million, representing nearly one fifth of the country’s total population, or 18.7 percent, highlighting the accelerating trend of China’s aging society, according to the National Bureau of Statistics; China’s population aged 65 and above is projected to reach 384 million (nearly one-third of the total population) by 2035 [2]. Concurrently, diseases such as type 1 diabetes and childhood obesity are becoming more prevalent worldwide [3,4], underscoring an urgent need for precise, personalized, and continuous health monitoring solutions.

In this context, health monitoring sensors, as the core tool for achieving proactive health management and early disease detection, have become increasingly important. It is worth noting that the “health monitoring” discussed in this article specifically refers to health monitoring for humans, which includes the collection of bioelectrical signals, monitoring of vital signs, and monitoring of biochemical parameters, etc. [5,6,7]. The current mainstream wearable and implantable sensors not only need to have high sensitivity and real-time feedback capabilities, but also need to meet multiple requirements, such as biocompatibility, long-term stability, human adaptability, and multi-signal acquisition. However, traditional manufacturing processes have significant limitations in terms of structural complexity, material diversity, and personalized customization, making it difficult to meet the development needs of the next generation of health monitoring sensors. Three-dimensional printing technology, as a breakthrough manufacturing method, enables high-precision construction of complex geometries, supports multi-material integration and personalized design, and meets the complex requirements of health monitoring sensors in miniaturization for body conformity, multi-material functional integration, multi-signal monitoring, and efficient batch production.

To ensure a comprehensive and up-to-date review, we conducted a systematic literature search across multiple scientific databases, including Web of Science, Wiley Online Library, Google Scholar, and Springer Nature. Key search terms included: ‘3D printing’, ‘additive manufacturing’, ‘sensor’, ‘wearable sensor’, ‘health monitoring’, ‘DIW’, ‘SLA’, and ‘DLP’. The literature was collected from the year 2000 onward, with a particular focus on high-impact original research and review articles from peer-reviewed journals published in the last five years. This emphasis on recent, high-quality publications ensures that this review captures the latest advancements and emerging trends in the field. This foundational search informed the scope and structure of this review.

Compared to existing reviews that primarily focus on technical principles or specific application scenarios [8,9], this review proposes a classification of 3D printing technologies according to their suitability for addressing diverse requirements in health monitoring sensors. Furthermore, we outline current challenges and future prospects, discussing how advanced technologies—including artificial intelligence, actuator, multi-material and cloud data—are poised to drive the evolution of next-generation health monitoring sensors. This review captures this rapid industrialization trend of 3D printing in health monitoring sensors by providing a timely, problem-driven analysis. In this context, a novel ‘point-line-area’ framework is proposed, categorizing 3D printing technologies according to their fundamental fabrication units. The distinct strengths of each approach in achieving conformal miniaturization, integrating diverse functional materials, enabling multi-parameter sensing, and supporting rapid, scalable manufacturing are systematically analyzed, helping researchers align the most suitable techniques with specific application needs. Furthermore, we incorporate an artificial intelligence perspective to address current challenges and outline intelligent, scalable solutions reflective of the technology’s accelerating industrialization.

**Figure 1:**
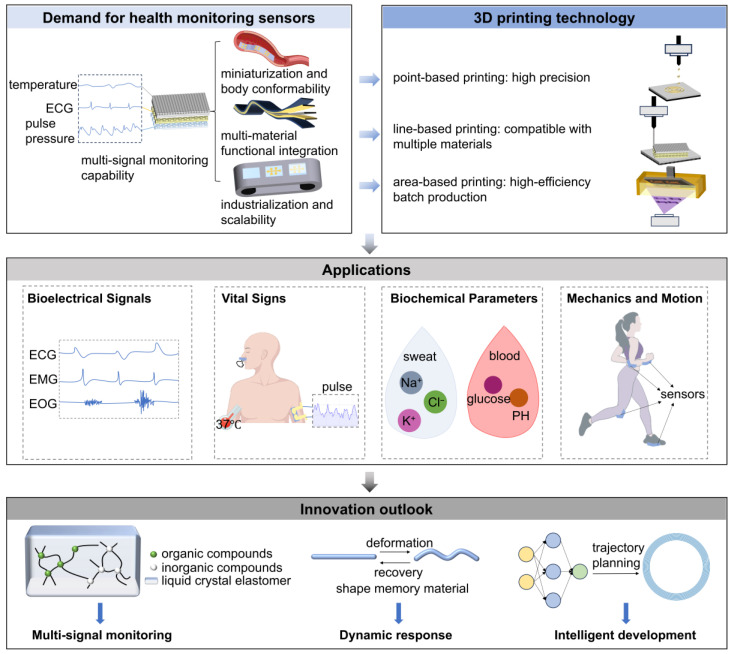
Created with BioGDP.com. [10] This graphical abstract provides a comprehensive overview of how 3D printing is enabling the development of next-generation health monitoring sensors. The paper begins by establishing the growing demand for advanced sensors. It then systematically analyzes how different 3D printing technologies, categorized by their fundamental fabrication unit, are uniquely suited to meet these needs, with practical applications discussed through research case studies. The review summarizes with an innovation outlook that discusses how the integration of advanced concepts, such as multi-functional materials, AI-driven design and other advanced techniques will drive the future of intelligent and personalized health monitoring.

In this article, as shown in Figure 1, the core requirements for health monitoring sensors are dissected first, and the unique advantages of 3D printing technology in addressing these requirements are analyzed as a whole. Then, by type of 3D printing fabrication unit, case studies are used to match different printing methods with the actual needs of health monitoring sensors. Finally, on the basis of a comprehensive review of the latest progress, we anticipate the future research directions of AI-driven design and multi-signal fusion to open up new ideas for the design and manufacture of the next generation of health monitoring sensors.

## 2. Requirements Analysis for Health Monitoring Sensors

The design and manufacture of health monitoring sensors need to satisfy increasingly diverse and stringent requirements to address the complex demands of modern medical diagnostics and personalized health management. This section analyzes four core requirements—miniaturization and conformability, multi-material integration, multi-signal monitoring capability, and scalability for industrialization—and highlights the unique advantages of 3D printing over conventional manufacturing approaches in fulfilling these demands.

(1) Miniaturization and Body Conformability

Wearable health monitoring sensors need to be miniaturized, flexible and lightweight [11] to fit the curves of the human body and ensure wearing comfort for long-term, burden-free monitoring of physiological signals. For implantable sensors, small size and biocompatibility are particularly important [12] to work stably in narrow or curved parts of the body while avoiding triggering immune rejection reactions.

3D printing is suitable for achieving layer-by-layer formation, combined with high-precision motion control systems and micron-level nozzles or laser beams, which can effectively control the feature size and reduce material waste. This capability allows the production of miniaturized, anatomically conformable sensor architectures [13,14,15,16] that are challenging to achieve with traditional techniques.

(2) Multi-Material Functional Integration

Material selection directly determines the functionality and safety of the sensor. The ideal material should have high sensitivity, long-term stability [17], good biocompatibility [18], and excellent mechanical properties [19], so as to ensure reliable data output in complex physiological environments. At the same time, different parts of the sensor require different materials and serve different functions. The parts used for signal transmission need conductive materials, the parts used for isolation need insulating materials, and the parts used for detection need sensitive materials, so the manufacturing of sensors has further demands for the simultaneous use of multiple materials.

3D printing is highly adaptable in terms of material selection and can process a wide range of materials, including polymers, metals, ceramics and composites [20,21,22]. It is also possible to precisely control the spatial distribution of different materials by changing the structure of the nozzles or increasing the number of nozzles to achieve multi-material integrated forming of the printed structure.

(3) Multi-signal Monitoring Capability

With the rise of personalized medicine and multi-parameter monitoring, the structure of sensors is becoming increasingly complex. Modern sensors often need to integrate multiple functional modules [23], such as temperature, pressure, and electrophysiological signal acquisition units, etc. Sensors made by traditional methods often fail to meet the urgent need for multi-dimensional data in modern health management and fail to provide users with more comprehensive health information [24].

3D printing, through layer-by-layer additive manufacturing, can achieve complex geometries that are difficult to process with traditional techniques, such as internal cavities, hollowed-out structures, and freeform surfaces, and can form highly complex structures in one piece, significantly enhancing design freedom and manufacturing flexibility [25,26,27]. Health monitoring sensors based on 3D printing can achieve personalized adaptation to different individuals through multi-material complex structure co-design, thereby enhancing the reliability of monitoring data and the user experience.

(4) Industrialization and Scalability

To transition from laboratory prototypes to clinical and consumer markets, sensor manufacturing must be efficient, cost-effective, and scalable [28]. Traditional manufacturing methods like screen printing, lithography, and template replication face limitations in producing the complex, customized, and biocompatible designs required for wearable and implantable sensors [29].

3D printing offers a solution by eliminating the need for molds and tooling, enabling rapid design iterations and patient-specific customization. For instance, the manufacturing process of prototypes is very fast. Newly designed sensors can be printed out and optimized within just a few hours [30,31,32,33].

3D printing is currently a developing trend, but issues such as post-processing of support structures, printing speed, and material costs remain obstacles to industrialization. The widespread industrialization of 3D printing for health sensors faces significant challenges, particularly concerning batch consistency and cost-effectiveness. The overall cost includes not just materials, but also the extensive post-processing and quality control steps necessary to meet the stringent requirements of industrialization and scalability. Specifically, achieving medical-grade consistency across a large batch of sensors remains a key hurdle, limiting its large-scale deployment compared to traditional manufacturing. There are still some research efforts aimed at improving costs and materials at present. For instance, Subedi et al. [34] reviewed several different vat-switching approaches for multi-vat stereolithography, significantly reducing vat-switching time and improving manufacturing efficiency. Additionally, Olawumi et al. [35] investigated material recycling in Fused Filament Fabrication, demonstrating potential for lowering material costs. These advancements suggest that 3D printing holds promising potential for future industrial applications.

3D printing technology uniquely addresses the multi-dimensional requirements of next-generation health monitoring sensors through its capabilities in micro-scale precision, multi-material integration, structural complexity, and scalable production [36,37]. These advancements suggest that while challenges remain, 3D printing holds immense promise for the future of personalized, high-performance health monitoring sensors, driving them towards smarter and more personalized directions, facilitating revolutionary changes to modern healthcare and health management.

## 3. Introduction to Printing Modalities: A Unit-Dimension-Based Classification

Significant advances have been achieved in the development of 3D-printed sensors for health monitoring. To systematically navigate the diverse landscape of additive manufacturing technologies and their match with functional requirements, this review adopts a novel classification framework based on the fundamental dimension of the fabrication unit: point, line, and area. This approach complements the established ISO/ASTM 52900 [38] standard by focusing on the inherent capabilities and application-oriented advantages of each modality, rather than solely on process origins.

Point-based fabrication methods construct structures through the sequential accumulation of discrete units, enabling high resolution and fine feature control. Line-based fabrication methods employ continuous material deposition, such as extruded filaments or ink streams, to build structures along digitally defined paths. This approach supports multi-material co-printing and complex geometrical fabrication, balancing design flexibility with functional integration. Area-based fabrication methods entire layer patterns or dynamic light fields at once, enabling rapid curing of full cross-sections or even volumetric segments. These methods achieve high throughput and facilitate the fabrication of intricate, often support-free architectures, making them suitable for batch production and complex sensor arrays. This dimensional framework not only clarifies the technical landscape but also bridges the gap between manufacturing capabilities and health sensor requirements, paving the way for intelligent design and personalized device fabrication.

### 3.1. Point-Based Printing

**Figure 2:**
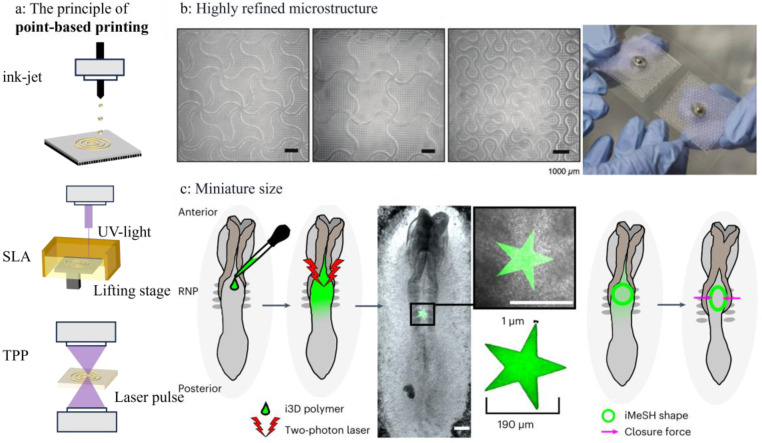
(**a**) 3D printing technology based on point formation. (**b**) The highly refined structure and physical diagram of the electrophysiological signal monitoring patch [47]. Copyright © 2024, The Author(s). Reprinted under the terms of the Creative Commons CC BY license. (**c**) Schematic diagram of two-photon printing technology and printing structures with tiny dimensions [49]. Copyright © 2024, The Author(s). Reprinted under the terms of the Creative Commons CC BY license.

Point-based printing technologies offer superior spatial resolution, typically ranging from nanometers to micrometers, making them ideal for fabricating miniaturized, high-precision components essential in advanced health monitoring systems. Despite relatively slower fabrication speeds, these methods are particularly suitable for applications requiring miniaturization and precision. Common techniques include inkjet printing, stereolithography (SLA), and two-photon polymerization (TPP) (Figure 2a).

Inkjet printing precisely deposits biological materials such as cells and biomolecules by controlling the nozzle. These nozzles can position and print out biomaterials layer by layer according to a designed three-dimensional model, thus forming the desired structure. It is more suitable for single-cell printing than for large-scale fabrication, with low printing efficiency and instability [39]. Therefore, to increase printing speed and variety of materials, multi-nozzle collaborative printing is added on the basis of single-nozzle inkjet printing [40,41]. SLA typically uses a laser as the light source to cure biological materials point by point. The stage moves up and down in the material pool, and with each layer cured, new material is added to the top, gradually forming a complete 3D structure [42]. Similarly to all point-based printing methods, it has the characteristics of high precision but low efficiency [43]. TPP utilizes focused femtosecond lasers to trigger nonlinear two-photon absorption in photosensitive materials, enabling ultra-high-resolution 3D printing at the sub-micron scale. It possesses excellent spatial control capabilities and flexibility in the fabrication of complex microstructures. This supreme resolution and excellent spatial control make TPP the preferred technique for manufacturing nano-scale structures [44].

The following cases exemplify how point-based printing addresses the stringent requirements of advanced, miniaturized sensing applications:

Electrophysiological Signal Monitoring: The high resolution of point-based printing is ideal for creating electrodes that conform to skin or tissue for monitoring bioelectrical signals, like electroencephalograms (EEG), electrocardiograms (ECG), electromyographs (EMG), etc., [45]. Zhang et al. [46] developed a stretchable organic electrochemical transistor (ISOECTs). They used multi-channel inkjet to print SEBS stretchable substrates, TAP buffer layers, Au electrodes, silver nanowires, PEDOT: PSS channels and ionic gel electrolytes layer by layer. The feature size of the fabricated ISOECTs can be as small as 100 μm, and the stretchability has reached 50%, which is used for the acquisition and processing of electromyography signals. Alsharif A. et al. [47] used SLA technology to print skin patches and fabricated negative molds with various microsphere patterns and snake—shaped microgrooves specifically for demonstrating ultra-fine micro-details (Figure 2b). Attaching it to the chest, around the eyeballs, arms and thighs can detect ECG, EOG and EMG.

Vascular Monitoring Sensors: The capability to print micro-features directly onto or as implantable devices is a key advantage. Herbert et al. [48] developed an implantable vascular electronic device that stacked capacitive pressure sensors using aerosol jet printing technology, with the most critical part being a PDMS dielectric layer with microstructure that enhances the sensor’s sensitivity and can detect continuous pressure changes. Vascular pressure, pulse rate and blood flow can be detected through the integration of a single sensor and multiple sensors.

Force Sensors: Point-based printing permits the creation of novel tools for fundamental biomechanical research at the microscale. Maniou et al. [49] used two-photon printing technology to print three-dimensional structures directly inside living embryos, being able to print star-shaped structures ranging from 1 μm at the tip to about 200 μm at the tip spacing (Figure 2c), which can reflect the mechanical force at which the neural tube closes by the degree of deformation.

Microfluidic and Microneedle Platforms: For biofluid analysis, point-based printing fabricates complex microfluidic architectures. Wu et al. [50] also developed a sweat microfluidic device using the SLA printing method, which contains a 200 μm deep microfluidic channel and has a complex structure and small size. It can calculate the concentration of copper ions in sweat by using the peak absorbance. Parrilla et al. [51] fabricated sharp hollow microneedle arrays using SLA printing technology. The open-circuit potential (OCP) between the working electrode and the reference electrode varies with the pH value. Therefore, after inserting the array into the human skin, the pH value in the human interstitial fluid can be calculated by detecting the OCP value. It is crucial for continuous monitoring of the wound condition or other medical conditions.

In summary, the application spectrum of point-based printing is vast, spanning from bioelectrical signal detection to vascular flow monitoring, from probing embryonic developmental forces to quantifying biomarkers in body fluids. The microscale dimensions and fine microstructures achieved unequivocally prove that point-based printing is uniquely capable of manufacturing high-resolution sensors that meet the critical demand for miniaturization and conformity in advanced health monitoring.

### 3.2. Line-Based Printing

Line-based printing technologies utilize continuous lines as the fundamental fabrication unit. This modality is distinguished by its unparalleled capability for multi-material integration and the creation of complex, functional three-dimensional structures, making it a cornerstone for advanced health monitoring sensors that demand material heterogeneity and customized geometries.

**Figure 3:**
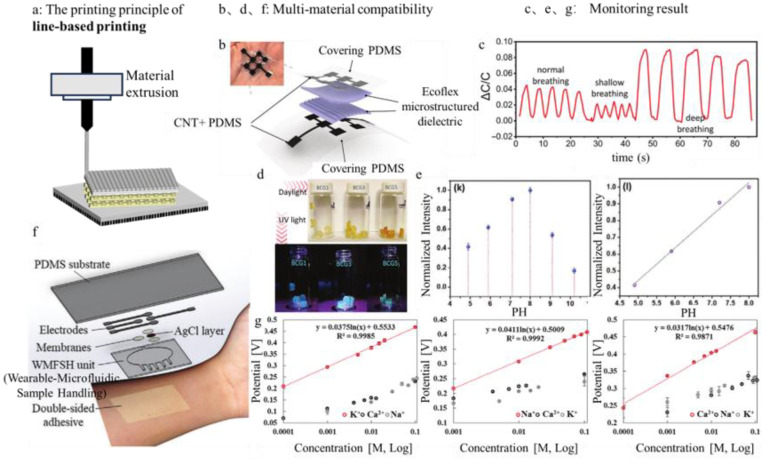
(**a**) Material extrusion. (**b**,**c**) Physical drawing and structural diagram of a capacitive pressure sensor manufactured by extrusion 3D printing, and capacitance changes under different breathing modes when it is placed on the abdomen [68]. Copyright © 2021, Wiley-VCH GmbH. (**d**,**e**) The preparation process of mixed hydrogels and their phenomena under different illumination, as well as the relationship between the fluorescence intensity of mixed hydrogels and pH value [72]. Copyright © 2024, The Author(s). Advanced Materials published by Wiley-VCH GmbH. (**f**,**g**) Structure diagram of the wearable patch and the relationship between the potential of the ion sensor and the concentration of the detected ion [73]. Copyright © 2021, Wiley-VCH GmbH.

Material extrusion, as defined by the ISO/ASTM 52900 standard (Figure 3a), encompasses two principal techniques: Fused Filament Fabrication (FFF) and Direct Ink Writing (DIW). These two methods are typical for material extrusion technology. FFF uses thermoplastic filaments while DIW employs extrudable inks.

FFF is a highly important and accessible technology, particularly for wearable health monitoring devices. Its strengths in low-cost customization, high accessibility [52] and rapid prototyping of supportive frames and housings are undeniable. It is a material extrusion process based on thermoplastic filaments, with common materials including polylactic acid (PLA) and acrylonitrile-butadiene-styrene (ABS) [53]. The filament is heated to its melting point, extruded through a nozzle, and deposited layer by layer along a programmed path, where it rapidly solidifies upon cooling, ensuring shape fidelity and strong interlayer adhesion. The reliability of FFF depends not only on melt flow during heating but also on controlled solidification during cooling. The post-deposition solidification process is influenced by both material properties, such as crystallization kinetics [54] and glass transition temperature [55], and process conditions. These factors collectively govern the structure and mechanical integrity of the printed part. In health monitoring sensors, FFF is commonly used to fabricate substrates, encapsulation housings, and support structures [56,57]. Its advantages include high-dimensional stability, mechanical robustness, and the ability to integrate conductive filaments (e.g., carbon fiber-reinforced PLA or conductive polymers) via co-extrusion or multi-material printing, enabling preliminary electrode or circuit fabrication and providing structural integration and physical protection.

**Figure 4:**
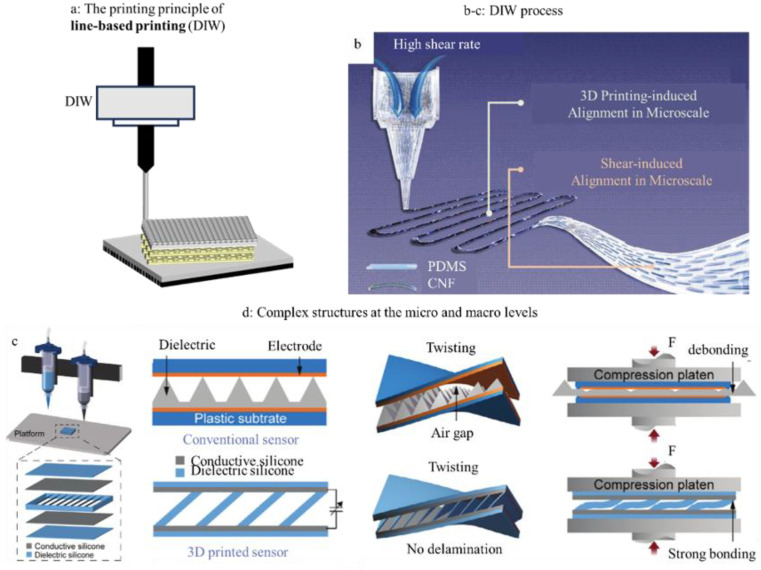
(**a**) DIW. (**b**) The manufacturing process of DIW and its directional complex structure at both micro and macro levels [74]. Copyright © 2024, Wiley-VCH GmbH. (**c**,**d**) The manufacturing process of DIW and the complex structure of the self-supporting inclined thin plate produced [75]. Copyright © 2024, The Author(s). Advanced Science published by Wiley-VCH GmbH. Reprinted under the terms of the Creative Commons CC BY license.

DIW, in contrast, employs extrudable, functional inks (Figure 4a), enabling precise spatial distribution of diverse materials—from conductive composites to biocompatible hydrogels—within a single device [58]. Health monitoring sensors often require the integration of multiple functionalities, such as sensitive physiological signal detection, mechanical adaptability, and efficient biocompatibility, which are distributed across distinct parts of the device, thus demanding heterogeneous material properties. With DIW, different ink formulations can be tailored and deposited within a single device based on functional needs. In sensing regions, conductive inks containing carbon nanotubes, graphene, or conductive polymers can be printed to construct highly responsive electrical pathways [59]; In structural or encapsulation layers, synthetic polymers can be selected for their combination of flexibility and mechanical robustness, ensuring device durability [60]; at the tissue-interfacing layer, natural polymers such as gelatin, sodium alginate, or hyaluronic acid can serve as biocompatible matrix materials, loaded with enzymes, cells, or growth factors to confer bioactivity and environmental responsiveness [61]. This on-demand ink customization not only aligns material properties with functional requirements but, combined with multi-nozzle systems, enables spatially ordered distribution of multiple materials and functional integration within three-dimensional architectures. Post-printing structures can be rapidly stabilized through ionic crosslinking, photopolymerization, thermal response, or chemical crosslinking, ensuring structural integrity and long-term operational stability under complex physiological conditions.

It is worth noting that DIW imposes specific rheological requirements on the ink formulation, key terms explanation can be found in Box 1. An ideal ink should exhibit shear-thinning behavior and rapid structural recovery [62] to ensure smooth extrusion and shape fidelity after deposition. Specifically, during the extrusion process, applied shear forces reduce the ink’s viscosity, facilitating flow through the nozzle. Upon cessation of shear, the material must rapidly rebuild its internal structure within milliseconds to seconds, characterized by a swift crossover of the storage modulus (G′) over the loss modulus (G″), leading to the formation of a transient gel. This viscoelastic solid-like state provides immediate structural self-support, effectively preventing collapse or spreading of the printed features. Long-term mechanical stability is typically achieved through post-deposition curing mechanisms such as photopolymerization, thermal crosslinking, or solvent evaporation, which further enhance G′ and consolidate the network. This staged rheological response enables DIW to fabricate complex 3D architectures, such as overhanging or porous structures [58], which are essential for high-sensitivity, stretchable, and conformal health monitoring sensors.

Box 1. **Key Rheological Terms for DIW Inks.**

TermExplanationShear-thinningA property where the material’s viscosity decreases under shear stress (e.g., during extrusion), allowing it to flow easily through the nozzle. Once the stress is removed, it thickens again.Storage Modulus (G′)Measures the solid-like behavior of a material; higher G′ indicates stronger structural rigidity and shape-holding ability.Loss Modulus (G″)Reflects the liquid-like behavior; when G″ > G′, the material flows.

**Figure 5:**
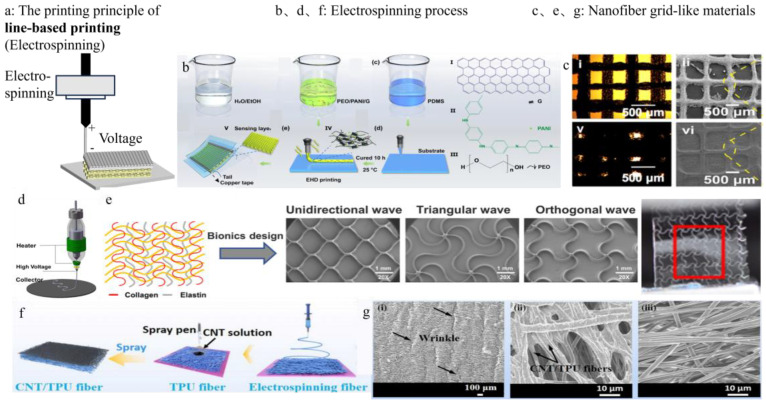
(**a**) Electrospinning. (**b**,**c**) The manufacturing process of electronic 3D printing technology and the structural characterization of the fabricated sensors [78]. Copyright © 2022, American Chemical Society. (**d**,**e**) Thin-film physical drawings and their results for detecting joint motion and heart beating [79]. Copyright © 2023, The Author(s). Reprinted under the terms of the Creative Commons CC BY license. (**f**,**g**) The manufacturing process of electrospinning and the structural characterization of the resulting materials [80]. Copyright © 2024, The Author(s). InfoMat published by UESTC and John Wiley & Sons Australia, Ltd. Reprinted under the terms of the Creative Commons CC BY license.

However, for implantable sensors, where direct and intimate contact with tissues and cells is required, the demands for miniaturization, feature resolution, and surface finish are far more stringent. The reliability of the product printed using material extrusion depends on the degree of adhesion between the lines, and the precision depends on the thickness of the lines [63]. To address this drawback, an electric field can be applied to the extruded filament, and a force can be applied, which is electrospinning (Figure 5a) [64] and near-field electrospinning. This method can control the thickness of the filamentous material extruded by the nozzle, improving the millimeter-level precision of extruded filaments to micron-level precision, and is suitable for manufacturing complex fiber network structures [65], constructing composite materials with specific functions, and enhancing the performance of health monitoring sensors by improving the performance of the materials. However, near-field electrospinning faces challenges in interlayer adhesion due to limited fusion between deposited fibers, which may compromise structural integrity [66]. Additionally, the process typically produces highly aligned fibers, resulting in anisotropic mechanical and electrical properties. While this anisotropy can be a limitation in some applications, it offers a distinct advantage in biomedical sensing: the aligned topography provides contact guidance that promotes directional cell attachment and growth [67].

In conclusion, the material extrusion technology demonstrates strong adaptability in the field of health monitoring sensors. By flexibly configuring and combining different types of “inks” or “filaments”, material extrusion can achieve a full material gradient design ranging from rigid support to flexible sensing, from insulation packaging to biological interfaces, fully meeting the requirements of sensors for multiple materials, multiple functions, and multi-level structures.

The versatility of line-based printing is exemplified across diverse sensing domains:

Respiratory and Cardiovascular Monitoring: Material extrusion enables direct printing of conformal sensors for respiratory assessment. Yi et al. [68] fabricated a capacitive pressure sensor. The PDMS support layer, the CNT+PDMS conductive layer and the Ecoflex porous grid pattern dielectric layer of the sensor were all made by material extrusion. When placed on the abdomen of the human body, the frequency and amplitude under different breathing modes could be reflected through the change in the sensor’s capacitance, realizing the monitoring of human respiratory activities (Figure 3b,c). In vitro detection responds to overall breathing patterns, while in vivo detection can specifically respond to the breathing patterns of a particular organ. Zhu et al. [69] directly extruded and printed flexible sensors of the appropriate shape on the extracorporeal breathing lungs of pigs to monitor the breathing patterns of the lungs. The main material of the sensor was a lithium chloride ion layer and polyacrylamide, which formed hydrogel ink and silica gel ink. Material extrusion could well adapt to the different characteristics of these two materials to achieve deposition.

Biochemical Sensing in Bodily Fluids: Line-based printing supports the integration of selective sensing chemistries for continuous biomarker monitoring. Adams et al. [70] used material extrusion to extrude conductive graphene into filaments and form patterned glucose concentration sensors on polyester film substrates. This study demonstrates the potential for future application in the daily blood sugar management of diabetic patients. Wu et al. [71] developed a microneedle patch based on material extrusion that can be used to regulate blood glucose levels in the body. For blood glucose detection, a unique bioink with sodium alginate and HAP was configured, and glucose needs to be filled in the needle tip to release insulin in response. This kind of microneedle patch omits the process of obtaining data through general sensor detection and directly provides corresponding treatments based on the detected signals.

Das et al. [72] used material extrusion to print hydrogel inks with different concentrations of carbon dots (CDs), creating a mixture of hydrogels with optical properties (Figure 3d,e). This method enhances the material’s toughness, resilience and adhesion, and can be used as a pH sensor to detect pH changes throughout the wound healing process. It also protects the wound from harmful UV radiation to prevent infection, promotes collagen synthesis and improves wound recovery efficiency.

Kim et al. [73] fabricated a wearable patch using material extrusion. The patch was stacked layer by layer, and the sensing unit chamber was based on an ion-selective membrane with Na^+^, K^+^ and Ca^2+^ corresponding carriers of polyvinyl chloride (PVC)-tetrahydrofuran (THF) (Figure 3f,g), which could be used to calculate the corresponding ion concentrations in sweat and achieve continuous monitoring of personal health parameters.

In material extrusion-based sensor fabrication, the printed materials generally fall into three main categories: (i) elastomeric matrices such as polydimethylsiloxane (PDMS), which offer flexibility, biocompatibility, and durability; (ii) hydrogel precursors, including sodium alginate, gelatin, and pluronic-based formulations, which enable soft, hydrated, and skin-conformal structures; and (iii) functional inks composed of conductive or bioactive fillers dispersed in printable binders. These can be loaded with additives such as carbon nanotubes (CNT), hydroxyapatite (HAp), or silver (Ag) nanoparticles for enhancing electrical performance. Furthermore, ion-selective membrane solutions, such as those based on valinomycin or neutral ionophores, can be precisely patterned to detect specific electrolytes in sweat, including Na^+^, K^+^, and Ca^2+^.

Motion and Activity Capture: The ability to print compliant, complex structures makes DIW ideal for motion sensors. Wang et al. [74] used DIW to directly print nanofiber CNF and PDMS composites with a layered structure, highly aligned macroscopically and highly oriented microscopically (Figure 4b). These layered nanocomposite sensors exhibit different local strain responses, thereby demonstrating multi-directional sensing capabilities. The three multi-directional sensors can record and compare the differences between the correct grip posture and the normal training posture of badminton players, thereby helping them improve their skills. Not only do athletes’ movement postures need to be monitored and corrected, but also the gait postures of ordinary people need to be improved through sensor detection. Zhu et al. [75] used DIW to fabricate a soft silicon-based capacitive sensor with a self-supporting inclined thin plate without support, which can effectively solve the problem of easy delamination of ordinary capacitive sensors (Figure 4c,d). Embedding it in the insole can be used for foot posture analysis, identifying abnormal pressure areas, and designing targeted intervention measures. The direct use of 3D printing to make electronic clothing is an emerging method that highlights personalization and can better match the flexibility of clothing than rigid wires. He et al. [76] used DIW to print circuits directly on clothing and various substrates to form flexible sensors, showing good adhesion and consistency. The movement of elbow and knee joints can be monitored by directly printing the joint sensor onto sleeves or pantyhose. In addition to the methods mentioned above for improving sensor sensitivity, Zhang et al. [77] can effectively enhance the sensor’s flexibility, elasticity and sensitivity by printing high-porosity silicone rubber foam through DIW technology, which can be used to detect weak pressure changes caused by pulse.

Motion and Activity Capture: Song et al. [78] fabricated the pressure sensor and chose electrospinning technology because it is more suitable for printing polymer materials embedded with functional nanoparticles or bioactive compounds (Figure 5b,c), which can effectively improve the sensor’s sensitivity. Without any signal processing, this sensor can detect radial artery pressure waves, and all three characteristic waveforms in the pulse wave can be clearly detected. Chen et al. [79] prepared an ultrafine fiber network through melt electrodeposition and fabricated a film with a thickness of 500 µm to form a flexible strain sensor, achieving a high degree of adhesion to soft tissues (Figure 5d,e). It can be applied to in vitro joint movement, muscle movement potential detection, heart beating detection, and so on.

Thermal Regulation: Wang et al. [80] used electrospinning technology to create TPU nanofibers with unique nanostructures, large specific surface area and high porosity, providing numerous binding sites for carbon nanotubes (CNTS), enabling CNTS to firmly bond with TPU nanofibers (Figure 5f,g) and serve as raw materials for temperature sensors. The multi-signal detection of health monitoring sensors has always been the focus of researchers’ exploration.

Line-based printing technologies provide a versatile platform for manufacturing health monitoring sensors that require multi-material integration, structural complexity, and tailored functional properties. While material extrusion (FFF and DIW) enables the direct fabrication of device architectures with embedded functionality, electrospinning enhances sensor performance through advanced material engineering. Collectively, these techniques support the development of sensors for respiratory, biochemical, kinetic, and thermal sensing, underscoring their critical role in advancing personalized health monitoring solutions.

### 3.3. Area-Based Printing

**Figure 6:**
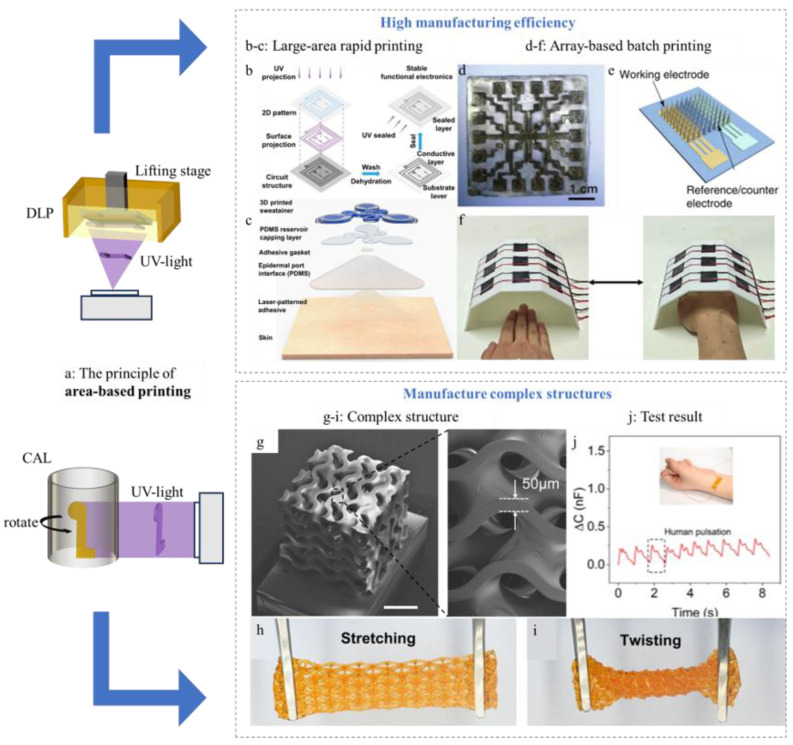
(**a**) 3D printing technology based on area formation. (**b**) Highly conductive hydrogel production process [93]. Copyright © 2024, Wiley-VCH GmbH. (**c**) DLP printed optical sensor structure diagram [94]. Copyright © 2023, The American Association for the Advancement of Science. Reprinted under the terms of the Creative Commons CC BY license. (**d**) DLP printed array type resistance strain sensor [95]. Copyright © 2020, Wiley-VCH GmbH. (**e**) Microneedle array sensor patch [97]. Copyright © 2021, The Author(s). Reprinted under the terms of the Creative Commons CC BY license. (**f**) A 3 × 5 sensor array capable of detecting temperature in two-dimensional space [96]. Copyright © 2024, Wiley-VCH GmbH. (**g**–**i**) Complex structures printed by VBP technology. (**j**) Pulse detection results of the EDL capacitance sensor printed by DLP [99]. Copyright © 2024, The Author(s). Reprinted under a Creative Commons Attribution-Noncommercial (CC BY-NC) license.

Area-based printing technologies, which employ two-dimensional planes or dynamic patterns as the fundamental fabrication unit, are renowned for their exceptional printing speed and ability to manufacture complex, high-resolution structures in a rapid layer-by-layer manner. This modality is ideally suited for the high-throughput production of sensor arrays and the fabrication of intricate device geometries that are challenging to achieve with other methods. Key techniques include Digital Light Processing (DLP) and Computed Axial Lithography (CAL) (Figure 6a), with the latter representing an advanced form of volumetric additive manufacturing that extends the concept of area-based fabrication.

DLP projects the shape of each slice of the model through a trough at one time to the bottom of the stage via a projector, and the material illuminated by the projection is immediately solidified into a solid part [81]. When all the layers are printed, the entire 3D object is complete. This printing method is much more efficient in forming than the previous two types, and it can be printed in array batches, ensuring that the manufacturing process is exactly the same. But the material must be chosen to have light-curing properties, so the material compatibility is not as good as that of material extrusion [82,83,84]. Moreover, the use of photoinitiators can raise biocompatibility concerns, as residual initiators may leach out and cause cytotoxicity [85]. The intensity and duration of light exposure also play critical roles: insufficient irradiation leads to incomplete curing, while excessive exposure may cause overheating or damage sensitive biomolecules [86]. These factors must be carefully optimized to ensure both structural integrity and biological safety in wearable or implantable sensors. Another area-based printing method is CAL, which is inspired by computed tomography (CT). This paper considers CAL as an extreme extension of area-based fabrication. Although its underlying principle belongs to volumetric additive manufacturing (VBP), its implementation still relies on the core mechanism of dynamic mask projection. During the process, the resin vat continuously rotates, while the projection system serially delivers two-dimensional patterns along the rotational axis. Through spatial accumulation of light energy, the polymerization threshold is reached at specific voxels, triggering localized curing. This process essentially synthesizes the projected patterns over time through controlled motion, approximating true volumetric fabrication. It breaks the traditional constraint in layer-by-layer printing that each layer must be stacked parallel to the others, and has been widely applied in fabricating complex structures, organoids, scaffolds, and vascularized constructs [87,88,89,90,91].

The most prominent feature of the area-based printing method is its high printing speed [92], which can significantly speed up the manufacturing process of health monitoring sensors to meet the demands of industrialization. And it is particularly suitable for array batch production because it can ensure that the sensor array is printed with exactly the same manufacturing process and environment, thus ensuring the consistency and accuracy of its performance.

The unique advantages of area-based printing are leveraged across diverse sensing applications:

High-Throughput Sensor Array Production: The parallelized nature of area-based printing makes it ideal for manufacturing sensor arrays with consistent performance. Zhang et al. [93] used DLP technology to print the desired personalized circuit structure quickly (Figure 6b). By sensing the phase signals of eye movement and encoding and decoding the direction of eye movement and the 26 English letters, communication with deaf and mute people can be achieved. Wu et al. [94] used the DLP method to rapidly print an optical sensor that can detect the chloride concentration in sweat over a large area (Figure 6c). This sensor can display different colors according to the detected chloride concentration. Xiao et al. [95] printed sensing layers made of carbon nanotube/elastomer composites directly on EA substrates based on DLP technology to form a 4 × 4 resistance strain sensor array (Figure 6d) that can detect human movement caused by bending. Extending one-dimensional detection to two-dimensional can add information such as position, which can reflect a person’s state more comprehensively. Weng et al. [96] used DLP to create a 3 × 5 sandwich array of sensors (Figure 6f) that can detect temperature in a two-dimensional space. Liu et al. [97] also developed a 9 × 9 microneedle array sensor patch based on DLP (Figure 6e), which can react with different concentrations of glucose to generate different current signals, ultimately enabling continuous monitoring of blood glucose concentration by detecting current magnitude. Tang et al. [98] used three types of hydrogels that respond, respectively, to pressure, temperature and pH stimuli as matrices, and combined them with periodically arranged air columns with specific reflection spectra to form sensors that can be implanted inside the brain. Among them, the periodically arranged gas column structure is formed by printing 25 cylindrical models at one time through DLP and then casting them. This can save time and ensure the consistent performance of the final formed sensors. The sensors can directly detect three physical quantities: pressure, temperature and pH. DLP technology can simultaneously print multiple sensors, meeting the requirement of using the health monitoring sensors as an array.

Complex Structural Fabrication for Enhanced Sensing: Area-based printing enables the creation of sophisticated architectures that enhance sensor functionality. Ge et al. [99] developed a highly conductive ion condensation, and for this material, DLP is an effective way to endow ion gels with complex structures such as octahedral truss structures and Gyroid structures (Figure 6g–i). So based on the DLP technology, a high-resolution, high-capacitance electronic double layer (EDL) capacitive sensor was developed that can detect tiny pressures, such as changes in a human pulse, and can clearly distinguish three typical main waves: shock waves, tidal waves, and relaxation waves. Peng et al. [100] used CAL technology to fabricate a solid particle conductive elastomer. This method can print a suspended structure without auxiliary support, avoiding the effect of subsequent removal of the support structure on the surface quality of the printed structure. It also has the ability to print nested structures in a single operation. It is also possible to fabricate composite structures that combine electronic and ionic conductors, widening the possibility of spatially arbitrary circuit deposition. Due to the microstructure of the copper foil, the capacitance of the sensor changes significantly when it is under pressure, which can detect human movement and be further applied to the athlete training data acquisition system to help athletes better understand the training situation and improve their technology.

Area-based printing technologies, particularly DLP and CAL, offer unparalleled capabilities in high-throughput sensor production and complex structural fabrication. While DLP provides exceptional speed and resolution for array-based manufacturing, CAL enables unprecedented geometric freedom for support-free, intricate sensor architectures. These technologies address critical needs in health monitoring for scalable production of consistent sensor arrays and the development of complex, multifunctional sensing platforms, positioning area-based printing as an essential modality for advancing toward intelligent, personalized health monitoring systems.

## 4. Current Challenges and Future Prospects

With growing emphasis on personalized medicine and wearable devices, there has been an accelerated demand for high-performance, multifunctional, and customizable sensors. Three-dimensional printing has emerged as a pivotal enabling technology for next-generation sensor manufacturing, offering unique advantages in miniaturization, material flexibility, structural complexity, and scalable customization.

As summarized in Table 1, point-based printing techniques enable sub-micron fabrication, meeting the requirements of micro- and nano-scale sensing units. Line-based approaches support multi-material co-printing and custom functional inks, making them suitable for constructing complex geometries and biocompatible structures. Area-based methods offer high printing efficiency, ideal for batch production of sensor arrays or large-area substrates.

**Figure 7:**
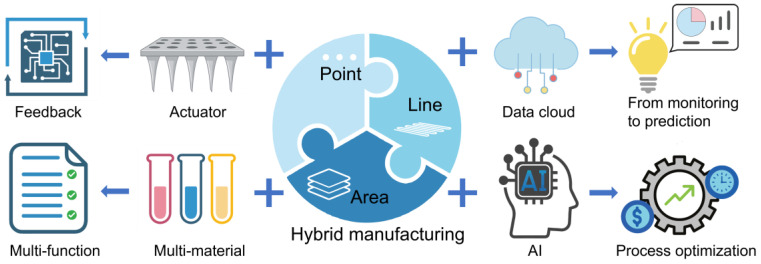
Future prospects. This schematic outlines how the integration of advanced technologies will empower the development of next-generation 3D-printed health monitoring sensors. The incorporation of actuators permits closed-loop feedback systems. Multi-material printing facilitates the integration of complex functions, while AI drives the optimization of sensor design and manufacturing. Data cloud enables a shift from passive health monitoring to proactive health prediction. The forward-looking strategy provides a pathway for creating intelligent, responsive, and highly customized sensors tailored for personalized healthcare. The graphical elements in Figure 7 were created with BioRender.com. We thank BioRender for providing the scientific icons used in this illustration.

Despite these strengths, several key challenges impede the widespread adoption of 3D printed health monitoring sensors, including spanning materials, manufacturing processes, and functional integration. This section outlines these challenges and proposes promising solutions, offering a roadmap toward intelligent, adaptive and sustainable sensor systems, through interdisciplinary integration. The future prospects are shown in Figure 7.

(1) Material Biocompatibility and Multifunctionality

Ideally, sensor materials should combine excellent biocompatibility, long-term stability, safe degradation characteristics, and the ability to respond to multimodal physiological signals, including electrical, mechanical, and chemical stimuli. However, current mainstream material systems still face limitations. Although conductive polymers and hydrogels exhibit good flexibility and biocompatibility, they are prone to performance degradation, degradation over time, or sensitivity drift during prolonged use [109,110,111,112], which restricts their application in implantable or long-term monitoring scenarios. For permanent implants, materials must be non-toxic, stable, and inert over time. In contrast, degradable materials (e.g., PLGA, gelatin) are designed to resorb after fulfilling their function. While advantageous for temporary sensing, their degradation products and kinetics must be carefully evaluated: acidic byproducts may cause local inflammation, and uncontrolled breakdown can compromise device integrity or tissue response. Thus, innovative material designs should not only focus on initial biocompatibility but also assess the biological impact of degradation throughout the implantation period.

Moreover, different 3D printing techniques vary in material compatibility: Point-based printing offers high precision, but the types of bio-ink available for use are limited [113]; Line-based printing supports multi-material fabrication, yet weak interfacial bonding between heterogeneous materials can compromise device reliability [114,115,116]; Area-based printing relies on photopolymerization, restricting material choices mainly to acrylate or epoxy-based resins, which often fail to meet requirements for biodegradability and low toxicity [117].

Potential solutions: Development of composite functional materials with stimuli-responsive and self-healing properties. For example, incorporating carbon nanotubes, graphene, or metal nanoparticles into liquid crystal elastomers or hydrogel matrices can endow materials with synergistic responsiveness to multiple stimuli such as temperature, pH, and strain [118]. What is more, the latest Dual-wavelength UV-curable 3D printing technology can be employed. This technology utilizes two different wavelengths of ultraviolet light to independently control different photochemical reactions, enabling the printing of multi-material structures with varying mechanical strengths. This significantly broadens the achievable mechanical performance range and improves the construction efficiency [119]. Based on the further development of printing technologies, such as Roll to Roll and Continuous Liquid Interface Production (CLIP), they will also promote the development of multi-material and multi-functional integrated sensors. Additionally, machine learning algorithms can be employed to establish predictive models that link “material composition–structure–performance,” accelerating the screening and optimization of novel composite materials, significantly shortening development cycles, and expanding the design space for high-performance biomaterials [120,121,122]. Elbadawi et al. [123] developed a pharmaceutical software that utilizes artificial intelligence machine learning technology (ML) to accelerate FFF printing and predict the printability of drug formulations. By using only the weighted scores of the materials in the formulation as input, they achieved a 76% accuracy in printability. Similar ML-driven material optimization can be applied to functional sensor inks, such as predicting conductivity-viscosity trade-offs in CNT/PEDOT: PSS composites for printed electrodes. This approach may accelerate the development of high-performance sensing materials.

(2) Efficiency–Accuracy Trade-off in Cross-scale Manufacturing

Health monitoring sensors typically require the integration of micrometer-scale sensing elements, such as electrodes and microchannels, with millimeter- to centimeter-scale packaging structures or flexible substrates. This imposes cross-scale requirements on manufacturing technologies. However, existing 3D printing methods struggle to simultaneously achieve high precision and high speed:

Point-based printing, such as two-photon polymerization (TPP), offers sub-micron resolution down to 100 nm [124], making it suitable for fabricating microstructures. However, the point-by-point scanning mechanism results in extremely low printing speed. For example, fabricating a microstructure of approximately 1.5 mm^3^ can take up to one hour, which limits scalability [101]. Line-based printing can construct three-dimensional structures at the centimeter scale. However, the nozzle diameter is typically larger than 100 μm, which restricts the ability to produce sub-micron features [102,103]. In contrast, area-based printing cures entire layers at once, with each layer solidified in just a few seconds, enabling high fabrication efficiency [104]. Furthermore, its resolution is generally limited to the micrometer scale, making it insufficient for manufacturing smaller-scale sensing units [125,126]. Furthermore, the complexity of the system and the compatibility issues of interfaces resulting from the integration of multiple processes also hinder the development of integrated manufacturing.

Potential solution: Develop a hybrid manufacturing platform that integrates the strengths of multiple printing techniques. A “zoning and layering” strategy can be adopted: Use point-based printing to fabricate high-precision micro-sensing structures; Employ line-based printing for functional multi-material integration and construction of complex 3D geometries; Utilize area-based printing to efficiently produce large-area substrates or encapsulation layers. Peng et al. [127] combined DLP technology with DIW technology and adopted a hybrid printing process. By leveraging the advantages of both DLP technology and DIW technology, they were able to fabricate embedded electronic components and stretchable strain sensors through a single print. Furthermore, integrating artificial intelligence to optimize printing parameters can enhance both printing speed and process stability [128,129,130]. Dai et al. [131] need to find the optimal combination of components for a biological ink that can print fibers with a diameter of 0.3 to 0.6 mm. They use AI to optimize and predict the results of all 81 possible combinations, and precisely screen out the parameter combinations that meet the requirements. This significantly simplifies the optimization process from hundreds of experiments to only a few validations, greatly improving the efficiency and repeatability of biological printing. This high-throughput screening strategy provides a paradigm for optimizing sensing materials to achieve target electrical-mechanical properties of printed sensors. Meanwhile, the modular manufacturing and post-assembly strategies are explored, where high-precision components and the main structure are manufactured separately and then assembled [132,133], taking into account both individualized and mass production requirements.

(3) Functional Integration and Intelligence

Next-generation health monitoring requires sensors capable of real-time sensing, adaptive response, and even self-repair. Current devices remain limited by signal cross-talk, environmental sensitivity, and insufficient long-term reliability [134,135].

Potential solution: Develop intelligent, responsive sensor systems. Use shape-memory polymers or stimuli-responsive hydrogels to fabricate sensors that can actively adjust their morphology or performance in response to changes in temperature, pH, or mechanical stress, enabling environment-adaptive sensing [136,137,138]; Incorporate self-healing materials that leverage dynamic covalent bonds or similar mechanisms to autonomously repair cracks or aged regions, thereby extending device lifespan [139]; Embed neural network algorithms in the data processing module to enable real-time signal decoupling, noise reduction, and pattern recognition across multiple channels, improving the accuracy and intelligence of information acquisition [140,141,142]. Lee et al. [143] designed a modular soft glove with enhanced tactile feedback and multi-modal sensing capabilities. Combined with machine learning algorithms, this intelligent glove cannot only detect delicate hand movements in real time, but also can achieve accurate object recognition and enhanced feedback, significantly enhancing the perception and communication of more comprehensive information. Such multimodal sensing and real-time feedback mechanisms exemplify the potential of embedded AI in 3D-printed health monitoring sensors for adaptive physiological monitoring.

(4) Sustainable and Scalable Development Paths

3D printing faces industrialization challenges due to limited batch-to-batch consistency. For high-volume production, scalable strategies should leverage the unique characteristics of each method to enable continuous, stable, and reproducible manufacturing through process standardization, automation, and real-time quality control.

Point-based printing should focus on parallelized manufacturing, employing strategies such as multi-focus systems or multi-nozzle arrays to enable simultaneous multi-point fabrication, thereby significantly improving printing efficiency and serving small-batch, highly customized personalized medical applications [144].

Line-based printing can be integrated with machine learning and real-time feedback control to establish a mapping model among “printing parameters, material properties, and sensing responses.” This enables dynamic optimization of extrusion rate, temperature, pressure, and other process parameters, as well as intelligent recognition of multimodal signals, paving the way for multifunctional sensing systems with real-time responsiveness [145,146,147,148].

Area-based printing should develop new biodegradable and non-toxic photopolymer materials that are environmentally friendly, promoting green manufacturing [149,150]. At the same time, explore the application of blockchain technology in the full life cycle management of materials and processes, establish a traceable quality control system, improve product consistency [151,152], accelerate regulatory approval through organizations such as the FDA (U.S. Food and Drug Administration), and promote the commercialization and implementation of high-performance sensor arrays. For example, COAPTIUM^®^ CONNECT (equipped with TISSIUM Light), a fully bioresorbable 3D-printed medical device jointly developed by U.S.-based 3D printing manufacturer 3D Systems and French medical technology company TISSIUM, received FDA approval in 2025 for 3D-printed peripheral nerve repair, offering clinicians an innovative therapeutic solution [153].

## 5. Conclusions

Three-dimensional printing achieves an effective balance among structural freedom, material diversity, and manufacturing efficiency by integrating the advantages of point-based, line-based, and area-based fabrication methods, providing strong support for cross-scale design, multi-functional integration, and personalized manufacturing of health monitoring sensors. The point–line–area framework accelerates the development of advanced health monitoring sensors by guiding optimal technology selection for specific applications. For personalized medical devices, point-based printing enables patient-specific, high-resolution designs; line-based methods support multi-material integration essential for wearable, real-time remote monitoring systems; and area-based techniques allow rapid fabrication of standardized sensor arrays for implantable or disposable devices. By aligning printing capabilities with functional demands, this paradigm streamlines the path from design to clinical deployment. In the future, with the deep integration of materials science, artificial intelligence, and advanced manufacturing, 3D printing will further drive the evolution of sensors toward intelligence, adaptability, and sustainability. This advancement will not only enhance device performance but also transform the paradigm of healthcare monitoring, opening broad prospects for personalized medicine, remote patient monitoring, and implantable medical devices.

## Figures and Tables

**Table 1:** Comparison of point-based, line-based, and area-based 3D printing technologies.

	Point-Based	Line-Based	Area-Based	References
Resolution	Nano to micrometer scale	Micrometer to millimeter scale	Micrometer scale	[42]
Printing Speed	Slow, point-by-point scanning (e.g., 1.5 mm^3^ takes ~1 h).	Moderate, suitable for centimeter-scale Structures.	Fast, entire layer cured simultaneously (seconds per layer).	[101,102,103,104]
Material Compatibility	Mainly photosensitive resins or hydrogels.	Capable of printing thermoplastics, hydrogels, composite pastes, bio-inks, etc.	Dependent on photopolymerizable materials; limited selection of biocompatible materials.	[105]
Biocompatibility	Requires specially developed biocompatible photosensitive ink.	Customizable bio-inks with natural/synthetic polymers, cells, or functional fillers.	Some photopolymerizable materials may have toxicity or produce harmful degradation byproducts.	[106]
Cost	Inkjet printing is cheaper. The printing technology involving special light sources is relatively expensive.	Material extrusion is the cheapest.	The printing technology involving special light sources is relatively expensive.	[107]
Scalability	Not suitable for large-scale devices.	Weaker interfacial bonding in multi-material printing.	Suitable for batch fabrication.	[108]

## Data Availability

Data availability is not applicable to this article as no new data were created or analyzed in this study.
